# Effects of oral maintenance chemotherapy and predictive value of circulating EBV DNA in metastatic nasopharyngeal carcinoma

**DOI:** 10.1002/cam4.2926

**Published:** 2020-02-23

**Authors:** Han Zhou, Tianzhu Lu, Qiaojuan Guo, Yan Chen, Mengwei Chen, Yansong Chen, Yingying Lin, Chuanben Chen, Liqin Ma, Yun Xu, Shaojun Lin, Jianji Pan

**Affiliations:** ^1^ Department of Radiation Oncology Fujian Cancer Hospital & Fujian Medical University Cancer Hospital Fuzhou China; ^2^ Fujian Provincial Key Laboratory of Translational Cancer Medicine Fuzhou China; ^3^ Laboratory of Biochemistry and Molecular Biology Research Fujian Cancer Hospital Fuzhou China; ^4^ Department of Clinical Laboratory Fujian Cancer Hospital & Fujian Medical University Cancer Hospital Fuzhou China

**Keywords:** EBV‐DNA, metastatic nasopharyngeal carcinoma, oral maintenance chemotherapy, overall survival

## Abstract

**Background/Objectives:**

Oral maintenance chemotherapy can effectively prolong overall survival (OS) in many types of metastatic cancer, but its role in metastatic nasopharyngeal carcinoma (mNPC) is unclear. In this study, the efficacy of oral maintenance chemotherapy in mNPC and the effectiveness of circulating tumor EBV‐DNA for screening patients were evaluated.

**Methods:**

Between June 2016 and December 2017, 141 patients with mNPC who received platinum‐based systemic chemotherapy were included (median follow‐up time, 21 months). Patients were classified into two groups according to the administration of oral maintenance chemotherapy. Plasma samples were collected before, during, and after treatment for the measurement of circulating EBV DNA.

**Results:**

The 2‐year OS was higher for patients who received maintenance chemotherapy than for patients without maintenance chemotherapy (78.9% vs 62.7%, *P* = .016). Patients with undetectable posttreatment EBV‐DNA after 4‐6 cycles of systemic chemotherapy (n = 73) had a higher 2‐year OS than that of patients with detectable EBV‐DNA (n = 68) (82.16% vs 51.45%, *P* = .001). For patients with undetectable posttreatment EBV‐DNA, OS was better for those with maintenance chemotherapy than for those without (86.7% vs 73%, *P* = .027). For patients with detectable posttreatment EBV‐DNA, maintenance chemotherapy did not improve outcomes (49.5% vs 55.4%, *P* = .824). The most common acute events were hematological toxicity, and all were tolerable and curable.

**Conclusions:**

Oral maintenance chemotherapy with S1 or capecitabine can improve OS in mNPC. Posttreatment EBV‐DNA was not only an independent prognosis factor for mNPC but also can screen out beneficiaries of maintenance chemotherapy.

## Graphical abstract

Oral maintenance chemotherapy is beneficial in many types of metastatic cancer. In this retrospective analysis, we provide evidence that it improves overall survival in metastatic nasopharyngeal carcinoma (mNPC). Furthermore, we identified circulating EBV DNA as an independent prognostic factor and found that patients with undetectable EBV‐DNA but not with detectable EBV‐DNA may benefit from maintenance chemotherapy.

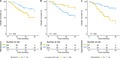

## 1 INTRODUCTION

The combined application of intensity‐modulated radiotherapy and chemotherapy has substantially improved outcomes in patients with nasopharyngeal carcinoma (NPC).^1^, ^2^ Distant metastasis is the main reason for treatment failure after radiochemotherapy, explaining about 20%‐30% of cases.^2^ In addition, 4%‐10% of patients with NPC develop distant metastases.^3^, ^4^ According to NCCN guidelines, chemotherapy with platinum‐based regimens is the first‐line treatment for patients with metastatic NPC (mNPC), with overall response rates of 70%‐80%.^3^ However, the 3‐year overall survival (OS) is only 33.7%‐60.7% for mNPC treated with first‐line chemotherapy.^5^, ^6^, ^7^, ^8^ Therefore, it is necessary to develop treatment strategies that can effectively improve survival in patients with mNPC.

Maintenance chemotherapy refers to continuous chemotherapy based on the effective control of tumors.^9^ To maximize tumor control, low‐dose and minimally toxic drugs are usually used for maintenance. Several studies have shown that maintenance chemotherapy can effectively control tumors and prolong OS in a variety of tumors.^10^, ^11^, ^12^ A variety of maintenance chemotherapy regimens have been evaluated for NPC. Molecular drugs, such as tyrosine kinase inhibitors, are ineffective in NPC,^13^, ^14^ but intravenous 5‐FU has shown good efficacy.^10^, ^15^ In recent years, maintenance chemotherapy with oral fluorouracil drugs (capecitabine and S1) have shown sufficient efficacies and relatively low toxicity in various cancers.^16^, ^17^, ^18^ Two studies have also shown that S1 in combination chemotherapy has antitumor effects in NPC.^19^, ^20^ However, it is unclear whether maintenance chemotherapy with oral fluorouracil can effectively control tumors and improve survival in NPC.

Furthermore, patient subsets expected to benefit from maintenance chemotherapy for NPC have not been identified. The selection of maintenance chemotherapy for NPC is currently based on imaging evaluations of the tumor response to treatment; however, the predictive value of these imaging‐based evaluations for the efficacy of oral maintenance chemotherapy is unclear. Circulating EBV DNA derived from tumors could be regarded as an archetypal circulating tumor DNA.^21^ The prognostic value of EBV‐DNA at pretreatment and posttreatment time points is clearly established in NPC.^22^, ^23^, ^24^ Changes in EBV‐DNA can reflect dynamic changes in tumor burden in NPC^25^ and are more effective than imaging for monitoring.^26^ Therefore, we hypothesize that EBV‐DNA levels after chemotherapy can be used to screen patients expected to benefit from oral maintenance in NPC.

We conducted a retrospective analysis to evaluate the efficacy of oral maintenance chemotherapy and to explore the utility of plasma EBV‐DNA for guiding the administration of maintenance chemotherapy in patients with mNPC.

## 2 MATERIALS AND METHODS

### 2.1 Patients

Hospital Review Board approval was obtained for this retrospective study. Patients with newly diagnosed mNPC (synchronous metastasis, SM) and patients with mNPC after radical treatment (metachronous metastasis, MM) at our institution between June 2016 and December 2017 were included. All patients had pathologically confirmed NPC at the primary site. The eligibility criteria were as follows: patients diagnosed with metastasis based on biopsy or radiologic imaging; patients accepted platinum‐based chemotherapy and underwent pretreatment or posttreatment plasma EBV‐DNA testing.

### 2.2 Treatment

All patients received platinum‐based systemic chemotherapy, including platinum plus gemcitabine and platinum plus paclitaxel. For patients with SM, 60‐70 Gy radiotherapy was applied to the primary nasopharynx and neck metastatic lymph nodes. Oligometastasis was defined as one to five metastatic lesions within two organs.^27^, ^28^ For patients with oligometastatic disease, local consolidative therapy (LCT) was suggested. LCT was defined as treatment with the intent to control all known sites of disease comprising surgery, radiotherapy, or radiofrequency ablation. For patients with a paralysis risk from spinal fractures or pain, radiotherapy was considered. For patients who achieved complete remission (CR), partial remission (PR), or stable disease (SD) after 4‐6 cycles of systemic chemotherapy, oral maintenance chemotherapy was administered with S1 or capecitabine. S1 was administered twice daily after a meal for 2 weeks followed by a 2‐week rest, at the following doses based on body surface area: <1.25 m^2^, 40 mg; <1.50 m^2^, 50 mg; and >1.50 m^2^, 60 mg. Capecitabine was administered orally at a dose of 1‐1.25 g/m^2^ twice daily in 4‐week cycles consisting of 2 weeks of treatment followed by rest period of 2 weeks. For patients with progressive disease, chemotherapy regimens were adjusted or changed.

### 2.3 Plasma EBV DNA levels

The patients underwent EBV‐DNA testing at the diagnosis of metastasis (pretreatment EBV‐DNA) and the end of systemic chemotherapy (posttreatment EBV‐DNA). Plasma EBV DNA concentrations were measured using a qPCR system, as described in a previous publication.^29^ In brief, plasma samples were subjected to DNA extraction using a Magnetic Beads Kits (EA20160201; PerkinElmer) and an automated nucleic acid extraction workstation (Pre‐NAT; PerkinElmer). A total of 450 μL of each plasma sample was used for DNA extraction. The exact amount was documented for the calculation of the target DNA concentration. A final elution volume of 60 μL was used to elute the DNA from the extraction column. Circulating EBV DNA concentrations were measured using a real‐time qPCR system to amplify a DNA segment in the BamHI‐W fragment region of the EBV genome. Data were collected using an ABI Prism 7500 Sequence Detector and analyzed using Sequence Detection System (version 1.6.3; Applied Biosystems). Results are expressed as copies of EBV genomes per milliliter of plasma. Multiple negative water blanks were included in every analysis.

### 2.4 Assessment and follow‐up

The patients underwent imaging evaluations every two cycles of systemic chemotherapy, including chest CT, abdominal CT/ultrasonography/MRI, bone ECT, head and neck MRI, or PET‐CT for the metastatic lesion and primary lesion. A response assessment was performed by imaging after every two courses of systemic chemotherapy, according to RECIST version 1.1. Bone metastasis was evaluated based on the response criteria for the practical management of osseous metastases proposed by Hamaoka et al^30^ For patients who underwent radiofrequency ablation, mRECIST was applied.^31^, ^32^ During the maintenance phase, all patients were assessed every 3 months in the first 2 years, every 6 months from years 2‐5, and annually thereafter. Toxicity was assessed weekly during radiotherapy and at every cycle of chemotherapy and was graded according to National Cancer Institute Common Terminology Criteria for Adverse Events version 4.0 (CTCAE 4.0).

### 2.5 Statistical analyses

OS was recorded as the day of diagnosis to the date of death or last follow‐up. Kaplan‐Meier survival analyses were used to estimate OS with log‐rank tests for the comparison of survival curves. Multivariate analyses with the Cox proportional hazards model with backward step‐down selection were used to identify significant independent prognostic factors. We used X‐tile software (version 3.6.1; Yale University) to find the optimal cutoff value of pretreatment EBV DNA. The parameters included in the Cox proportional hazards model were age (≤45 vs >45 years), gender (male vs female), cycles of systematic chemotherapy (<4 vs ≥4), maintenance chemotherapy (no vs yes), pre‐EBV‐DNA level, disease status (SM vs MM), oligometastasis (no vs yes), LCT (no vs yes), posttreatment EBV‐DNA (undetectable vs detectable), and imaging examination (CR/PR vs SD/PD). All statistical tests were two sided, and *P* < .05 was considered statistically significant. Statistical analyses were performed using SPSS (IBM version 18.0) and R version 3.6.1 (http://www.r-project.org).

## 3 RESULTS

### 3.1 Patient's characteristics and survival

**Figure 1:**
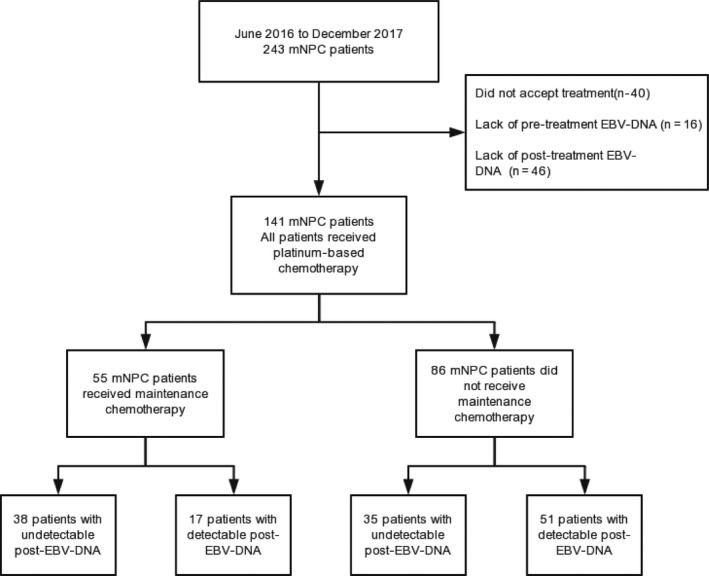
Flow chart

From June 2016 to December 2017, 243 patients with mNPC were diagnosed at our center. A total of 40 patients were excluded because they did not receive treatment and 62 patients were excluded due to the lack of pretreatment or posttreatment plasma EBV‐DNA information (Figure 1). Finally, 141 patients were included in this study. After or during chemotherapy, 43 (30.5%) patients received LCT for metastatic foci. Additionally, 55 (39.0%) patients received maintenance chemotherapy after platinum‐based chemotherapy. The median number of cycles of maintenance chemotherapy was 13 (range: 2‐31). Other clinical characteristics are summarized in Table 1. The median follow‐up time for the whole cohort was 21 months (range: 1‐36 months). In the follow‐up period, 41 (29.1%) patients died, and the 2‐year OS for the whole group was 70.4%.

**Table 1:** Clinical characteristics of 141 metastatic nasopharyngeal carcinoma patients

Covariate	N (%)	Maintenance chemotherapy		
		No (n = 86, 61.0%)	Yes (n = 55, 39.0%)	*P*
Gender				.468
Male	124 (87.9%)	77 (89.5%)	47 (85.5%)	
Female	17 (12.1%)	9 (10.5%)	8 (14.5%)	
Age(years)				.963
≤45	74 (52.5%)	45 (52.3%)	29 (52.7%)	
>45	67 (47.5%)	41 (47.7%)	26 (47.3%)	
KPS				.442
<90	33 (23.4%)	22 (25.6%)	11 (20%)	
≥90	108 (26.6%)	64 (74.4%)	44 (80%)	
Disease status				.528
Synchronous metastasis	79 (56.0%)	50 (58.1%)	29 (52.7%)	
Metachronous metastasis	62 (44.0%)	36 (41.9%)	26 (47.3%)	
Oligometastasis				.460
No	67 (47.5%)	43 (50%)	24 (43.6%)	
Yes	74 (52.5%)	43 (50%)	31 (56.4%)	
Pre‐EBV‐DNA (copies/ml)				.397
Median (Min, Max)	8.5 × 10^3^(0‐3.91 × 10^7^)			
≤760	38 (27.0%)	21 (24.4%)	17 (30.9%)	
>760	103 (73.0%)	65 (75.6%)	38 (69.1%)	
Chemotherapy cycles				.002
<4	14 (9.9%)	14 (16.3%)	0 (0)	
≥4	127 (90.1%)	72 (83.7%)	55 (100%)	
Local consolidative therapy				.045
No	112 (79.4%)	73 (84.9%)	39 (70.9%)	
Yes	29 (20.6%)	13 (15.1%)	16 (29.1%)	

### 3.2 Maintenance chemotherapy improves the OS

**Figure 2:**
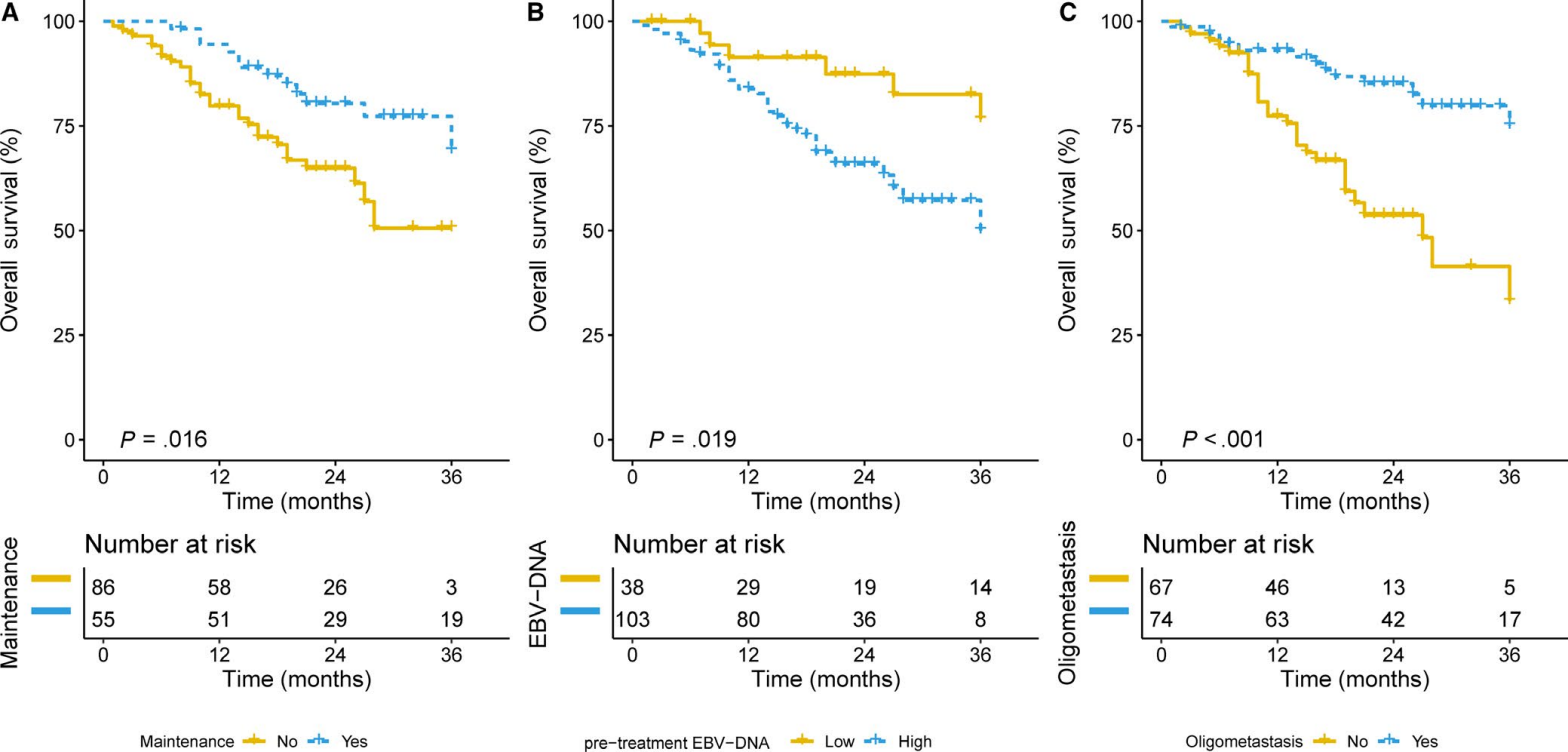
The Kaplan‐Meier curves of OS in mNPC patients: (A) OS for patients with or without maintenance chemotherapy; (B) OS for patients with high or low level of EBV‐DNA at pretreatment; (C) OS for patients with oligometastasis or multiple metastasis

A survival analysis showed that patients who received maintenance chemotherapy had a higher 2‐year OS than that of patients without maintenance chemotherapy (78.9% vs 62.7%, *P* = .016) (Figure 2A). To investigate the prognostic value of pre‐EBV‐DNA, 760 copies/mL was identified as the optimal cutoff value. Patients with low pre‐EBV‐DNA levels (<760 copies/mL) had a significantly higher 2‐year OS than that of patients with relatively high pre‐EBV‐DNA levels (84.6% vs 64.28%, *P* = .019) (Figure 2B). Kaplan‐Meier survival analyses showed that oligometastasis was a favorable factor (83.5%% vs 51.0%, *P* < .001) (Figure 2C). Other factors were not related to prognosis in patients with M1 NPC. A detailed summary of these results is provided in Table 2.

**Table 2:** Univariate and multivariate analysis of variables for metastatic nasopharyngeal carcinoma patients

Covariate	Univariate analysis		Multivariate analysis	
	2‐y OS (%)	*P*	HR (95%CI)	*P*
Gender				
Male	60.9	.327		
Female	70.3			
Age (years)				
≤45	68.5	.584		
>45	70.4			
KPS				
<90	57.5	.300		
≥90	71.9			
Disease status				
SM	66.1	.245		
MM	75.6			
Oligometastasis				
No	52.5	<.001	1.0	
Yes	83.5		0.30 (0.16‐0.59)	<.001
Pre‐EBV‐DNA (copies/ml)				
≤760	52.5	.019	1.0	
>760	83.5		2.24 (0.92‐5.48)	.075
Chemotherapy cycles				
<4	85.6	.618		
≥4	87.3			
Maintenance chemotherapy				
No	62.7	.016	1.0	
Yes	78.9		0.50 (0.25‐0.98)	.044
LCT				
No	66.2	.224		
Yes	80.8			

Abbreviations: MM, metachronous metastasis; SM, Synchronous metastasis; pre‐EBV‐DNA, pretreatment EBV‐DNA; LCT, local consolidative therapy.

After adjusting for gender, age, cycles of systemic chemotherapy, LCT, maintenance chemotherapy, pretreatment EBV‐DNA, disease status (SM vs MM), and oligometastasis (no vs yes), a multivariate analysis showed that maintenance chemotherapy and oligometastasis are independent prognostic factors for OS in mNPC (HR = 0.50, *P* = .044; and HR = 0.30, *P* < .001).

### 3.3 Post‐EBV‐DNA can predict beneficial effects of maintenance chemotherapy

**Figure 3:**
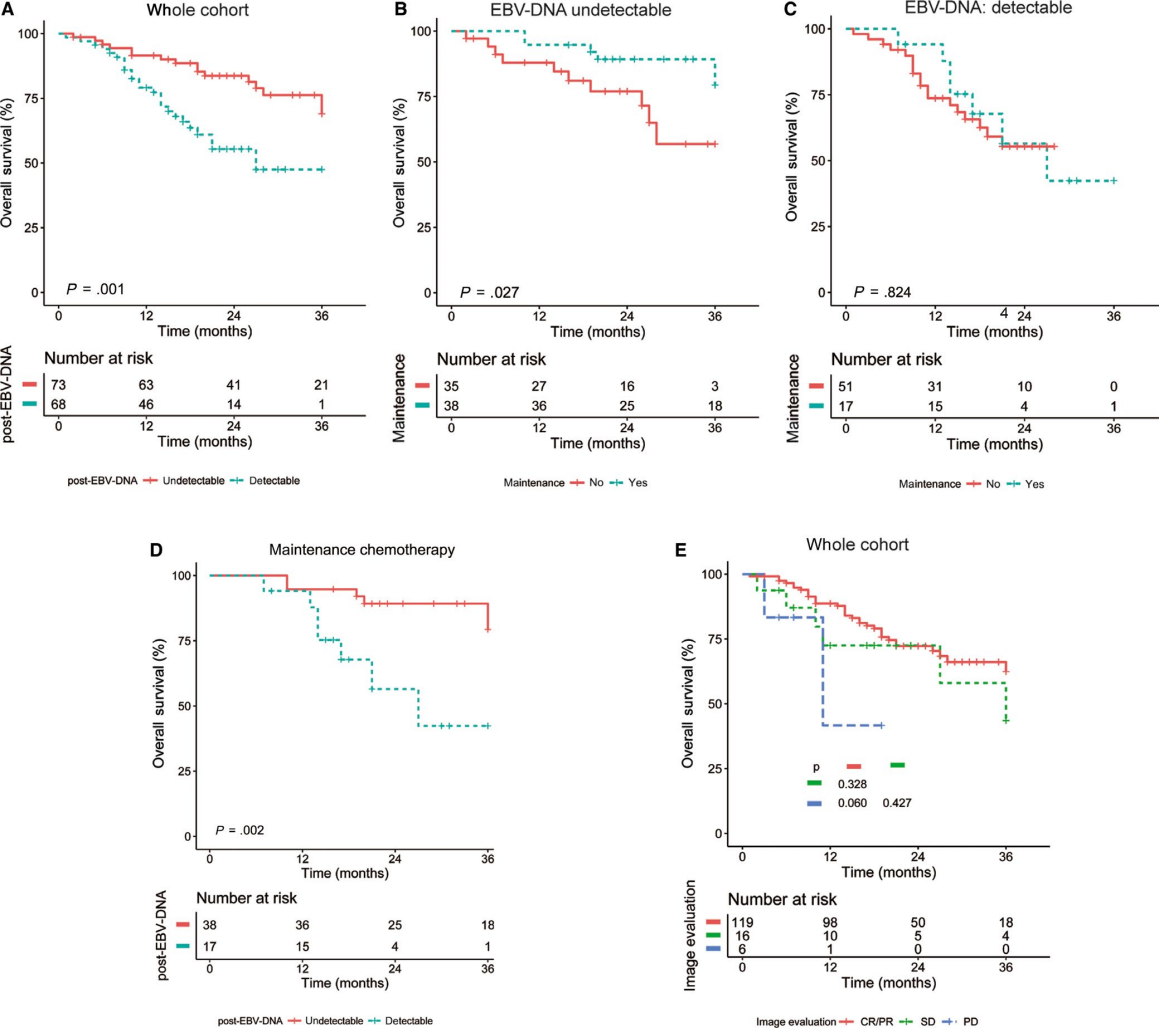
The Kaplan‐Meier curves of OS in mNPC patients: (A) OS for patients with undetectable or detectable EBV‐DNA at postsystematic chemotherapy; (B) OS for patients with or without maintenance chemotherapy in the cohort with undetectable EBV‐DNA at postsystematic chemotherapy; (C) OS for patients with or without maintenance chemotherapy in the cohort with detectable EBV‐DNA at postsystematic chemotherapy; (D) OS for patients undetectable or detectable EBV‐DNA in the cohort with maintenance chemotherapy; (E) OS for patient with different treatment response according image evaluation

After systemic chemotherapy, among 141 total cases, plasma samples from 73 patients had undetectable EBV‐DNA. A survival analysis showed that patients with undetectable posttreatment EBV‐DNA after systematic chemotherapy had a higher 2‐year OS than that of patients with detectable EBV‐DNA (82.16% vs 51.45%, *P* = .001) (Figure 3A). For patients with undetectable post‐EBV‐DNA, those who received maintenance chemotherapy had a better OS than that of patients without maintenance chemotherapy (86.7% vs 73%, *P* = .027) (Figure 3B). For patients with detectable posttreatment EBV‐DNA, the OS did not differ between patients with maintenance chemotherapy and without maintenance chemotherapy (49.5% vs 55.4%, *P* = .824) (Figure 3C). Among patients receiving maintenance chemotherapy, a survival analysis showed that OS was higher for those with undetectable posttreatment EBV‐DNA than those with detectable posttreatment EBV‐DNA (86.7% vs 49.5%, *P* = .002) (Figure 3D).

According to RECIST, 4 patients achieved CR, 115 achieved PR, 16 achieved SD, and 6 exhibited progressive disease. Survival analyses showed that radiologic evaluation did not affect the OS (Figure 3E). Among 115 patients with CR/PR, 51 (42.8%) patients with CR/PR had detectable posttreatment EBV‐DNA. Among 16 patients with SD, 5 (38.1%) had undetectable posttreatment EBV‐DNA. All patients with PD (6/6, 100%) had detectable posttreatment EBV‐DNA. A multivariate analysis confirmed that maintenance chemotherapy (HR = 0.48, 95% CI: 0.23‐0.97, *P* = .041), oligometastasis (HR = 0.45, 95% CI: 0.24‐0.86, *P* = .015), and detectable posttreatment EBV‐DNA (HR = 2.33, 95% CI: 1.16‐4.65, *P* = .017) were prognostic factors for OS in mNPC (Table 3).

**Table 3:** Multivariate analysis of OS by posttreatment EBV‐DNA and maintenance chemotherapy adjusting for other predictors in metastatic nasopharyngeal carcinoma patients

	Multivariate analysis	
	HR (95%CI)	*P*
Gender (male vs female)		
Age (≤45 vs >45)		
Chemotherapy cycles (<4 vs ≥4)		
Maintenance chemotherapy (NO vs YES)	0.48（0.23‐0.97）	.041
Local consolidative therapy (NO vs YES)		
Post‐EBV‐DNA (undetectable vs detectable)	2.32（1.16‐4.65）	.017
Oligometastasis (NO vs YES)	0.45（0.24‐0.86）	.015
Imaginative examination (CR/PR vs SD/PD)		

### 3.4 Toxicity of maintenance chemotherapy

The main toxicities were hematological‐related events of grade 1‐2, and only seven patients developed grade 3 toxicities (12.7%). Treatment interruption secondary to disease progression was observed in 17 (30.9%) patients. No grade 4 adverse events were recorded. There was no treatment‐related death. Toxicities during the maintenance setting are documented in Table 4. Long‐term side effects were not analyzed owing to the relatively short survival time.

**Table 4:** Toxicity of during maintenance chemotherapy (n = 55)

Toxicity	During maintenance therapy				
	Grade 0	Grade 1	Grade 2	Grade 3	Grade 4
Anemia	44	8	2	1	0
Neutropenia	17	17	16	5	0
Thrombocytopenia	41	6	5	3	0
Liver dysfunction	44	7	2	2	0
Gastrointestinal reaction	47	5	0	3	0
Mucositis	53	1	0	1	0
Hand‐foot syndrome	53	1	0	1	0

## 4 DISCUSSION

Metastasis is the primary cause of treatment failure in NPC.^2^ For patients with metastasis, it is important to optimize treatment strategies to improve the survival and disease control. Oral maintenance chemotherapy has satisfactory results in many kinds of diseases.^16^ Our results suggested that oral S1 or capecitabine significantly improve the OS for patients with mNPC. Furthermore, we found that posttreatment EBV‐DNA was not only an independent prognostic factor for mNPC but also a robust and crucial biomarker to identify patients who will benefit from maintenance chemotherapy. In brief, maintenance chemotherapy significantly improves OS for patients with undetectable posttreatment EBV‐DNA but not for patients with detectable posttreatment EBV‐DNA.

We found that the 2‐year OS of patients with mNPC with maintenance chemotherapy was significantly better than that of patients not receiving maintenance chemotherapy (78.9% vs 62.7%, *P* = .016). Although maintenance treatment with intravenous 5‐FU for mNPC has shown good results and tolerable toxicity,^10^ the outcome of oral maintenance chemotherapy with S‐1 or capecitabine in patients with mNPC has not been reported. Intravenous maintenance has various disadvantages, including inconvenience and pain. Safe and convenient maintenance regimes are worth exploring. A retrospective study has shown that the oral fluorouracil analog tegafur can effectively reduce the rate of distant metastasis in patients with NPC with high metastatic risk.^33^ Maintenance chemotherapy with S1 and capecitabine in a variety of metastatic cancers effectively improves the OS and has tolerable toxicity.^34^ Our results are consistent with these previous studies. Maintenance chemotherapy was generally tolerable, except in four patients who withdrew due to grade 3 mucositis after S‐1 (1/55, 1.8%) and grade 3 gastrointestinal side effects (3/55, 5.4%). Our data suggest that oral maintenance chemotherapy is a treatment strategy for mNPC, and its efficacy should be confirmed by clinical trials. Furthermore, the efficacies of maintenance chemotherapy with S1 and capecitabine should be compared and the duration of maintenance therapy should be optimized, among other issues that should be resolved in future studies. Currently, three clinical trials of oral maintenance chemotherapy in mNPC are ongoing (NCT02460419, NCT02878889, and NCT02944708), and these are expected to provide additional insight into the efficacy of maintenance therapy.

A key result of our study was that EBV‐DNA after systemic chemotherapy could be used to screen patients expected to benefit from maintenance chemotherapy. In particular, maintenance chemotherapy may not be sufficient for patients with detectable posttreatment EBV‐DNA. Maintenance chemotherapy is administered after the tumor has been effectively controlled.^9^ However, tumor control based on imaging evaluations may not accurately reflect the state of the tumor in vivo.^35^ Plasma EBV‐DNA can better reflect the tumor state and burden, because it is released from NPC cells into the blood circulation.^21^ Even when disease control is observed based on imaging, the detection of EBV‐DNA in plasma indicates that a large number of tumor cells are present in the body, and oral maintenance therapy may not be sufficient. Our results also show that patients with undetectable posttreatment EBV‐DNA may benefit substantially from maintenance chemotherapy. However, imaging evaluations cannot be used to assess prognosis in mNPC and to identify patients who could benefit from maintenance chemotherapy. In a variety of tumors, including NPC, undetectable levels of circulating tumor DNA in the plasma at the end of treatment suggest better survival.^21^, ^36^, ^37^ Accordingly, an undetectable level of EBV‐DNA after systemic chemotherapy could be a useful biomarker for screening individuals who may benefit from maintenance chemotherapy. Of course, this prediction requires additional evidence, especially from clinical trials.

In our study, patients with oligometastasis had better outcomes than those of patients with multiple metastases (83.5% vs 52.5%, *P* < .001). In many kinds of cancers, oligometastasis is an independent prognostic factor, including NPC.^38^, ^39^, ^40^, ^41^ Some studies have shown that after systemic therapy, patients with oligometastasis can reach more than 5 years of progress‐free survival.^42^, ^43^ Oligometastasis is recognized as an early stage of cancer progression, at which point the invasiveness is still weak, as evidenced by the low number of metastases and a limited number of organs affected.^44^, ^45^ Thus, oligometastasis can be seen as a vital indicator for chemotherapy and LCT.^28^ LCT did not improve survival in our study, probably owing to the small number of patients receiving LCT (29/141, 20.6%). For patients with oligometastasis, oral chemotherapy maintenance combined with LCT treatment after disease control (undetectable EBV‐DNA status) may be a powerful and effective treatment strategy, with the potential to cure mNPC.

Despite the promising outcomes of this research, several limitations should be addressed. First, this was a retrospective, single‐center analysis and according selection biases could not be avoided. Future multicenter and prospective studies are needed to determine whether maintenance therapy can improve OS in patients with mNPC. Second, EBV‐DNA clearance cannot be efficiently calculated owing to the low frequency of EBV‐DNA testing in our retrospective study. Finally, the data were derived from an academic cancer center in an endemic area. The findings should be reproduced and the generalizability to other cancer centers should be determined.

## 5 CONCLUSIONS

Oral maintenance chemotherapy with S1 or capecitabine has the potential to improve the OS for patients with mNPC. Posttreatment EBV‐DNA was an independent prognostic factor for mNPC with systemic chemotherapy also a marker for treatment decisions. For patients with detectable posttreatment EBV‐DNA, maintenance chemotherapy was not sufficient. If our findings are validated in other external studies or clinical trials, they provide an effective treatment strategy for mNPC and promote the use of EBV‐DNA to guide the use of maintenance chemotherapy.

## CONFLICT OF INTEREST

There are no conflicts of interest to declare.

## AUTHOR CONTRIBUTIONS

All authors contributed to data analysis, drafting or revising the article, gave final approval of the version to be published, and agree to be accountable for all aspects of the work.

## Data Availability

The datasets used and/or analyzed during the current study are available from the corresponding author on reasonable request.
